# A convenient synthesis of γ-functionalized cyclopentenones

**DOI:** 10.1186/1860-5397-1-11

**Published:** 2005-10-07

**Authors:** Nour Lahmar, Taïcir Ben Ayed, Moncef Bellassoued, Hassen Amri

**Affiliations:** 1 Laboratoire de Chimie Organique & Organométallique, Faculté des Sciences, Campus Universitaire 2092-Tunis, Tunisie; 2 Laboratoire de Synthèse Organométallique, 5 mail Gay-Lussac, Neuville sur Oise, 95031 Cergy-Pontoise Cedex, France

**Keywords:** Nef reaction, conjugated addition, nitroalkanes, 1,4-diketones, functionalized cyclopentenones

## Abstract

The synthesis of γ-functionalized cyclopentenones was carried out in a few steps, starting firstly with the preparation of nitroketonic intermediates **2**, which were readily transformed into 1,4-diketones using the Nef conversion. The intramolecular cyclization of the γ-diketones **3** in a basic medium, led to the functionalized cyclopentenones **4**.

## Introduction

The cyclopentenones are considered as an important class of compounds because they are present in a large variety of natural products and in many important biologically active compounds [1–6] such as prostanoids, jasmonoids, rethrolones, methylenomycins and allylrethrones. In this paper, we wish to describe a new synthetic procedure for the preparation of γ-functionalized cyclopentenones **4** *via* a conjugate addition of nitroalkanes to α,β-unsaturated ketones **1** leading to the nitroalkanes derivatives **2** which may be converted into their γ-diketone homologues **3** using Nef reaction. The intramolecular cyclization of 1,4-diketones **3** led to the corresponding cyclopentenones **4**.

## Results and discussion

**Scheme 1:**
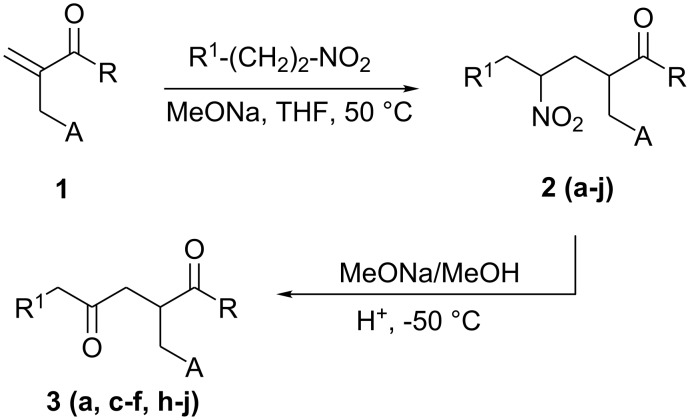
Synthesis of nitroalkanes derivatives **2** and their corresponding 1,4-diketones **3**.

As previously reported, the 1,4-addition of primary nitroalkanes [7–12] to functionalized α,β-unsaturated ketones was considered as an appropriate method to prepare multifunctional nitroketonic compounds. These intermediates are important in organic synthesis because the nitro group can be converted into other useful groups such as amines or carbonyls.[13–14] Treatment of α,β-unsaturated ketones **1**[15] with primary nitroalkanes was achieved by refluxing the mixture at 50°C in THF using sodium methoxide as base (Scheme 1), resulting in full conversion and yielding the Michael adducts **2** (Table 1).

**Table 1:** Synthesis of nitroalkanes derivatives **2** and their corresponding 1,4-diketones **3**

**A**	**R**	**R^1^**	**1,4-Nitroketone**	**Yield (%)**	**1,4-Diketone**	**Yield (%)**

CO_2_Me	Me	H	**2a**	65	**3a**	67
CO_2_Et	Me	H	**2b**	62	**3a**	64*
CH_2_CO_2_Et	Me	H	**2c**	61	**3c**	53*
Ph	Me	H	**2d**	76	**3d**	68
CO_2_Me	Ph	H	**2e**	68	**3e**	64
CO_2_Me	Me	Me	**2f**	61	**3f**	63
CO_2_Et	Me	Me	**2g**	62	**3f**	62*
CH_2_CO_2_Et	Me	Me	**2h**	61	**3h**	48*
Ph	Me	Me	**2i**	76	**3i**	65
CO_2_Me	Ph	Me	**2j**	70	**3j**	63

*This compound undergoes transesterification reaction.

Various synthetic methods were developed to obtain 1,4-diketones [16–20] since these compounds were valuable intermediates in the synthesis of cyclopentenone ring system. Obviously the most straightforward route was the Nef reaction. The transformation of the nitro derivatives to carbonyls by the formation of the nitronate anion in the presence of sodium methoxide followed by its addition to concentrated sulfuric acid at -50°C, allowed the formation of the 1,4-diketones **3** in fair to good yields (Table 1).

**Scheme 2:**
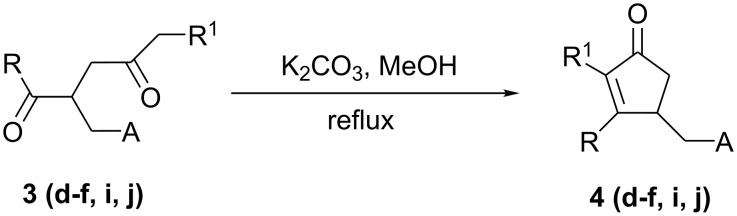
γ-Functionalized cyclopentenones **4**.

During recent years, some cyclopentenones derivatives were known to exhibit biological and pharmaceutical properties and have been widely studied [21–23]. In order to contribute to the synthesis of this family of products, we present in this work a strategy of preparation of a new γ-functionalized cyclopentenones **4**. Thus, we showed that intramolecular cyclization of the functionalized 1,4-diketones **3** could be conducted in the presence of anhydrous potassium carbonate (K_2_CO_3_) in the refluxing methanol to afford the pure cyclopentenones **4** with satisfactory yields (Table 2, Scheme 2).

**Table 2:** γ-Functionalized cyclopentenones **4** prepared

**Cyclopentenones**	**A**	**R**	**R^1^**	**Time (min)**	**Yield (%)**

**4d**	Ph	Me	H	90	72
**4e**	CO_2_Me	Ph	H	75	70
**4f**	CO_2_Me	Me	Me	80	65
**4i**	Ph	Me	Me	60	85
**4j**	CO_2_Me	Ph	Me	65	73

## Conclusion

A convenient procedure has been developed for the synthesis of γ-functionalized cyclopentenones **4** using the reaction between nitroalkanes and α,β-unsaturated ketones **1** to afford γ-nitroketonic intermediates **2** which may be converted into their γ-diketones homologues **3** using Nef reaction. The intramolecular cyclization of diketones **3** leads to the corresponding cyclopentenones **4**. The easy workup procedure and short reaction steps, impart greater merit of this methodology over those existing.

## Experimental

All reactions are carried out under a nitrogen atmosphere. Unless otherwise noted, all products are purified before use. ^1^H-NMR spectra (300 MHz) and ^13^C NMR spectra (75 MHz) were recorded on Bruker AC 300 MHz spectrophotometers using CDCl_3_ as solvent. Chemical shifts (δ) are given from TMS (0 ppm) as internal standard for ^1^H-NMR and CDCl_3_ (77.0 ppm) for ^13^C NMR. Flash chromatography was done on Merck grade 60 silica gel (230–400 mesh) using mixtures of hexane and ethyl acetate as eluent.

### Synthesis of nitroketones (2): General procedure

To a mixture of nitroalkanes (32 mmol) and sodium methoxide (32 mmol) in anhydrous THF (20 mL) was added vinylketone **1** (a-j) (8 mmol), the solution was stirred for 13 h at 50°C, the mixture was treated with H_2_O and extracted with Et_2_O (3 × 20 mL). The organic layer was washed with brine and dried (MgSO_4_). The solvent was removed to leave an oil which was purified by column chromatography on silica gel.

#### 3-Acetyl-5-nitrohexanoic acid methyl ester (2a)

Purified by column chromatography (hexane/AcOEt, 7/3). ^1^H NMR (300 MHz, CDCl_3_) δ 1.55 (d, 3H, *J* = 6.7 Hz), 2.29 (s, 3H), 2.37–2.79 (2 m, 4 H), 2.92 (m, 1H), 3.67 (s, 3H), 4.55 (m, 1H); ^13^C NMR (75 MHz, CDCl_3_) δ 19.8, 29.8, 34.4, 36.2, 44.7, 52.0, 81.6, 171.4, 208.4. HRMS Calcd C_9_H_15_NO_5_: 217.0950. Found: 217.0932.

### Preparation of functionalized 1,4-diketones (3): General procedure

At the solution of sodium (7.5 mmol) in 15 mL of methanol, was added at room temperature the nitro intermediate **2** (5 mmol). After one hour, a solution of H_2_SO_4_ (3 mL) in 15 mL of methanol at -50°C was added. The mixture was treated with H_2_O, the methanol was evaporated, and the residue was extracted with dichloromethane. The organic layer was washed by a solution of NaOH 1% and a solution of NaCl and dried (MgSO_4_). The solvent was removed to leave oil, which was purified by column chromatography on silica gel.

#### 3-Acetyl-5-oxohexanoic acid methyl ester (3a)

Purified by column chromatography (hexane/AcOEt, 7/3). ^1^H NMR (300 MHz, CDCl_3_) δ 2.15 (s, 3H), 2.28 (s, 3H), 2.43; 2.65 (ABd, 2H, *J_AB_* = 16.5 Hz, *J^A-H^* = 7 Hz, *J^B-H^* = 6.6 Hz), 2.60; 2.93 (ABd, 2H, *J_AB_* = 18.1 Hz, *J^A-H^* = 8 Hz, *J^B-H^* = 5.5 Hz), 3.36 (m, 1H), 3.67 (s, 3H); ^13^C NMR (75 MHz, CDCl_3_) δ 29.2, 29.8, 35.0, 42.9, 44.4, 51.9, 172.0, 206.2, 209.5. HRMS Calcd C_9_H_14_O_4_: 186.0892. Found: 186.0877.

### Preparation of γ-functionalized cyclopentenones (4): General procedure

To a solution of 1,4-diketone **3** (5 mmol) in MeOH (10 mL) was added 1 equivalent of K_2_CO_3_, the mixture was bring to reflux during one hour. After workup, the product **4** was purified by column chromatography on silica gel (EtOAc/hexane).

#### 4-Benzyl-3-methylcyclopent-2-enone (4d)

Purified by column chromatography (hexane/AcOEt, 8/2). ^1^H NMR (300 MHz, CDCl_3_) δ 2.09 (s, 3H), 2.26–2.59 (m, 2H), 2.71 (m, 1H), 3.21 (m, 2H), 5.92 (s, 1H), 7.17–7.28 (m, 5H); ^13^C NMR (75 MHz, CDCl_3_) δ 19.4, 37.0, 39.0, 48.0, 126.3, 128.4, 128.8, 129.8, 139.6, 177.6, 211.0. HRMS Calcd C_13_H_14_O: 186.1045. Found: 186.1029
